# Exploring reflective 'critical incident' documentation of professionalism lapses in a medical undergraduate setting

**DOI:** 10.1186/1472-6920-9-44

**Published:** 2009-07-15

**Authors:** David Hodges, John C McLachlan, Gabrielle M Finn

**Affiliations:** 1 School for Health, University of Durham, Thornaby, Stockton-on-Tees, UK

## Abstract

**Background:**

Measuring professionalism in undergraduate medical students is a difficult process, and no one method has currently emerged as the definitive means of assessment in this field. Student skills in reflection have been shown to be highly important in the development of professional behaviours. By studying student reflections on lapses in professional judgement, recorded as 'critical incidents', it is possible to explore themes which are significant for the development of professional behaviour in an undergraduate setting.

**Methods:**

We examined critical incident reporting combined with optional written student reflection as a method for exploring professionalism in undergraduate medical students. 228 students split between Year 1 and 2 of one academic year of undergraduate medicine were studied retrospectively and a grounded theory approach to analysis was employed.

**Results:**

This year generated 16 critical incident reports and corresponding student reflections, all of which were considered. In addition to identifying the nature of the critical incidents, 3 principal themes emerged. These were the impact and consequences of the report having been made, student reactions to the events (both positive and negative), and student responses regarding future actions.

**Conclusion:**

This study indicates that unprofessional behaviour can be identified and challenged by both the faculty and the students involved, and suggests that positive behavioural changes might be made with the aim of preventing future occurrences. We provide a low cost approach of measuring and recording professional behaviour.

## Background

Measuring professionalism in undergraduate medical students is a difficult process, and no one method has currently emerged as the definitive means of assessment in this field [1]. Recent literature reviews have highlighted the lack of current evidence when measuring attitudes related to professionalism in medical education [2] and have proposed 88 methods in the past 25 years [3]. This study examines critical incident reporting combined with voluntary student reflection within a UK medical school as a method for exploring professional behaviours and attitudes in a cohort of pre-clinical undergraduate medical students.

### Definition of Critical Incidents

Here we define 'critical incidents' for undergraduate medical students in lapses of professional standards or failures of diligence which in a clinical setting might have adverse effects on patients.

The advantages of critical incident reporting were first published by Rhoton, documenting the study of anaesthesiology residents in a US teaching hospital during a 5 year period in the 1980s [4,5]. These studies found that only 1% of negative behaviour related to areas of professionalism, and that there was a link between resident conscientiousness and unprofessional behaviour. Further to this, critical incident reporting was found to permit early intervention in unacceptable behaviour [6]. Incident reporting forms in recent studies demonstrate the use of areas of fixed concern, derived from professional standards the faculty feel students should exhibit, as a method to assess professionalism [7,8]. Critical incident reporting can be used as grounds for dismissal if continuing unprofessional behaviour is observed [7]. In the University of California, San Francisco, critical incident reports formed part of the medical student performance evaluation given on graduation if two or more reports had been issued during undergraduate study [7]. Outside of medical education, critical incident reporting remains an important aspect of improving patient safety and is used in all NHS trusts to monitor adverse events [9].

Student reflective skills have been seen to be highly important in the development of professional behaviours [10]. By studying student reflections on critical incident behaviour, the development of good professional behaviours from reflection could be explored. Its introduction could provide tools for students to examine their own behaviour and attitudes, to consider the reasons why incidents occurred, and to allow solutions to be developed for preventing such incidents in the future.

## Methods

Existing literature was reviewed to look for current evidence in this field. Medline was searched using keywords of 'professionalism' 'critical incident' 'incident reporting' 'medical school' and 'undergraduate'. Further manual searches within three leading journals (*Medical Teacher, Medical Education and Academic Medicine*) were done to ensure all relevant papers were considered.

This study used an unrestrictive method of incident reporting to record unprofessional behaviour utilising expert opinion by a process of connoisseurship [11]. Experienced staff members reviewed all incident reports and made decisions based on knowledge and personal experience as to whether the reports were valid and primarily focused on unprofessional behaviour. Unprofessional behaviour was defined by any act which broke the School code of conduct, which is based on the GMC Duties of a Doctor [12,13]. Students were given a copy of the report prior to a meeting with staff to discuss it, and invited to submit a reflective response using a structured form.

The study group was 228 undergraduate medical students in Years 1 and 2 of a U.K. medical school. The study material was all critical incident reports issued during the timeframe of a single academic year. The exclusion criteria were reports that did not refer to professional behaviour and incomplete reports. Framework analysis was done using a process of thematic analysis, and the data collected was analysed using a grounded theory approach [14]. All analysis took place at the end of the study period. Initial multiple readings of the transcripts with hand annotation was performed, before incorporating the data into word processor format for further study. All three authors analysed the data independently, with comparative discussion of findings performed to reach consensus and promote reliability of results.

The data used in this study was obtained from routinely collected information regarding student assessment and was not initially intended for the purpose of research. Subsequently, the information obtained proved, we believe, to be of generalisable interest [15]. Ethical approval from the University Ethics Committee for the use of educational research data was obtained, on condition that the data was anonymous and could not be linked to the individuals concerned. As a result, individual student consent was not obtained. All student data was anonymised, identifiable comments were excluded, and no identifiable harms arose from this study being conducted.

## Results

Over the study period, 16 critical incident forms were completed by members of faculty from a cohort of 228 students. Of these 2 were excluded following connoisseur review as they failed to meet the criteria set. The 14 forms related to 9 students, some of whom had multiple forms. Of the 14 incidents forms, 7 had a reflective response.

Seven elements of professionalism were raised by staff. These were poor communication, unexplained absence, record keeping, meeting deadlines, positive commitment to studying, honesty, and patient confidentiality. Students did not always recognise the same areas of professionalism as a concern within their reflections.

The following major themes emerged from analysis of the transcripts.

### Impacts and consequences

All students who reflected on the incidents considered the impact and consequences of their actions. There was recognition of how the student was involved.

Extract 1: *'This affected me and my studies.' *(Student A)

There was also recognition that members of staff, patients and other students may also have been affected. Students described the impact that their actions had on others, what burdens may have been placed upon them and what additional work, if any, the student had created for them.

Extract 2: *'It affected me and the people at my placement. I was not as useful and productive as I could have been...it affected my colleagues at the placement as they may have had to perform a greater share of the work that would have otherwise been the case.' *(Student B)

Extract 3: *'I did not attend my sessions in which I appreciate my clinical tutor has given up valuable time to come in' *(Student C)

The ideas of wasting the time of others and failure to complete their own work emerge from the explanations. Students are also concerned about how the incident may have changed perceptions of themselves and other students.

Extract 4: *'my patient may have been let down and lost respect for the responsibility of medical students' *(student A)

The concern of student concentrates on patient perception of the student which has been affected in a negative manner.

### Reflections

Identification of the responsibilities of a medical student is a necessary step to remediation. These reflections on the incidents fell into two sub themes: those of acceptance and those of rejection.

### Acceptance Reactions

With acceptance reactions, students demonstrated the recognition of fault in themselves, showed guilt and remorse for their actions and acknowledged a lack of knowledge could be the cause. Students included clear statements of acceptance.

Extract 5: *'I fully accept that my behaviour has been unacceptable'*

*'My failure to provide explanations and consult with the tutor is unacceptable' *(Student C)

Students also show the ability to describe how the event has affected them emotionally and their intended future response.

Extract 6: *'I feel very bad that I let this happen and it certainly won't happen again'*

*'I now realise that it was very serious and should not even have considered it' *(student D)

There is also student acceptance of blame which had been previously unrecognised.

Extract 7:*'For some reason it did not occur to me that the same principles applied in the lecture theatre' *(student D)

### Rejection reactions

Not all students in the study felt the reports were an accurate description of the event which occurred, and described aspects which they felt were out of their control.

Extract 8: *'...haven't received the test results from the medical centre'*

*'I was falsely led to believe...' *(Student E)

Denial of the accuracy of the event also occurred, and students disputed the incident. Students felt that their actions have been misinterpreted by the staff and that their actions were not unprofessional. These rejection reactions could also be seen to be both defiant and possibly complacent.

Extract 9: *'I was not listening to music during lectures' *(Student E)

Some responses do not deny that the event occurred, but reflected a denial of the significance of the event which occurred. Students did not always agree that what was reported constituted a critical incident or problem with professionalism.

Extract 10: '*No event, I missed a few important lectures*.' (Student A)

A minority of students recognised that their behaviour was unprofessional from the view of staff involved, but believed that their actions were justified due to the circumstances surrounding the issue.

Extract 11: *'I worked most of the holiday and believe this time would have been necessary regardless'*

'due to lack off work earlier in the year that I needed to catch up'

*'... but I believe that the alternative would have hindered my revision' *(Student A)

### Responses

Students frequently identified further actions, which in turn related to whether they had made acceptance or denial responses. Acceptance responses included remediation in the form of apologies:

Extract 12: *'I will write a personal letter of apology' *(Student C)

Extract 13: *'I take full responsibility for my actions and sincerely apologise to both X and Y, whose lectures I enjoy very much.' *(Student E)

Apologies can also relate to the theme of impact and consequences, with students being concerned with the perceptions staff may have of them in light of the incident. By apologising, students may hope to prevent this perception of them.

Rejection responses include suggestions for further actions by others, which could be constructive. Students feel that their experiences could be used to make practical changes to the course, and would as such be of benefit to their fellow students.

Extract 14: *'...instead of having three lectures on one topic, the student should be given a book to study with a certain time period. Then at the end of this time period there will be a review lecture to ensure everyone has understood.' *(Student F)

## Discussion

The approach of having critical incident reporting forms associated with reflective documents appears to have the possibility of promoting student reflection as a necessary first step to remediation of inappropriate behaviours. Suggesting practical steps to avoid the repeat of the situation in the future shows potential progression through the reflective cycle, with the hope that future behaviour will follow the changes the student suggests they could make. Even having such a system in place may serve as a promoter of good behaviour or an inhibitor of adverse behaviour, since students know that the critical incident form will be placed in their file. The evidence of Papadakis et al. suggests that such incidents can serve as a predictor of future behaviour, and an organised system of collection of such data could facilitate future progression decisions, or simplify the task of providing references or Dean's Letters.

The biggest barrier to gaining a positive outcome for faculty and student occurred when no reflective response was written by the student. In future use of the critical incident reporting, making reflection a compulsory aspect of the incident becomes an attractive proposition. An absence of reflection only has a negative value in assessing the student's professional behaviour, whereas student reflection can have either positive or negative value.

The curriculum of medical schools already incorporates reflection as part of summative assessment for many different taught subjects, and the 'Report and Reflect' system could be used in a similar manner. Establishing expected outcomes for reflection and having procedures in place for students who fail to meet the outcomes would allow for a structured assessment process for students involved in critical incidents. As a summative assessment it would also have formative value, the student would learn what is expected in future as a result, and would be able to change their professional behaviour as a result.

We acknowledge that there are limitations to the approach used. No evidence of data saturation was observed in the responses, and the study needs to be extended. Since critical incidents are rare events, extended periods of time will be required to build up a full analysis of causes and responses. The cohort of students involved in this study was small at 228, and the timeframe of single academic year was short. Involving multiple centres and increasing the timescale to the extent of 5–10 years, with further follow-up studies of students once working as doctors, would allow for improved reliability and could give evidence of predictive validity.

The utility of this approach can also be considered [16]. Reliability is dependent upon having staff with sufficient knowledge and experience to assess the reports. The educational impact should be positive, which can be seen when reflective practice improves future professional behaviour. Staff acceptability may relate to the time involved, with each report taking on average 90 minutes to complete in this study. Issues of validity relate to the GMC requirements for student behaviour, and for medical school monitoring of such behaviour.

## Conclusion

This study shows that by using a 'Report and Reflect' system of incident reporting, a strong platform can be created from which lapses in professional behaviour within the student body and be recorded and challenged. It has been seen that student reflection on critical incidents could promote positive changes in professional behaviour. Further to this an absence of reflection, or the inability of a student to respond adequately to events, might be a future indicator of unprofessional behaviour. Critical incident reports are enhanced when combined with student reflection.

## Competing interests

The authors declare that they have no competing interests.

## Authors' contributions

JM was responsible for the study design and construction. All authors worked equally in analysis of the data and generation of results. DH drafted the manuscript which was read, edited and approved by JM and GF.

## Pre-publication history

The pre-publication history for this paper can be accessed here:



## References

[B1] Shrank WH, Reed VA, Jernstedt GC (2004). Fostering Professionalism in Medical Education A Call for Improved Assessment and Meaningful Incentives. J Gen Intern Med.

[B2] Jha V, Bekker HL, Duffy SRG, Roberts TE (2007). A systematic review of studies assessing and facilitating attitudes towards professionalism in medicine. Medical Education.

[B3] Lynch DC, Surdyk PM, Eiser AR (2004). Assessing professionalism: a review of the literature. Medical Teacher.

[B4] Rhoton FM, Barnes A, Flashburg M, Ronail A, Springman S (1991). Influence of Anesthesiology Residents' Noncognitive Skills on the Occurrence of Critical Incidents and the Residents' Overall Clinical Performances. Academic Medicine.

[B5] Rhoton FM (1994). Professionalism and Clinical Excellence among Anesthesiology Residents. Academic Medicine.

[B6] Rhoton FM (1990). A new method to evaluate clinical performance and critical incidents in anaesthesia: quantification of daily comments by teachers. Med Educ.

[B7] Papadakis MA, Osborn EH, Cooke M, Healy K (1999). A strategy for the detection and evaluation of unprofessional behavior in medical students. University of California, San Francisco School of Medicine Clinical Clerkships Operation Committee. Academic Medicine.

[B8] Ainsworth MA, Szauter KM (2006). Medical Student Professionalism: Are We Measuring the Right Behaviors? A Comparison of Professional Lapses by Students and Physicians. Academic Medicine.

[B9] National Patient Safety Agency NHS, London (2004). Seven steps to patient safety. An overview guide for NHS staff.

[B10] Friedman BD, Davis MH, Harden RM, Howie PW, Ker J, Pippard MJ (2001). AMEE Medical Education Guide No: 24: Portfolios as a method of student assessment. Medical Teacher.

[B11] Misch DA (2002). Evaluating Physicians' Professionalism and Humanism: The Case for Humanism "Connoisseurs". Academic Medicine.

[B12] General Medical Council Publications, London 2006 Good Medical Practice.

[B13] General Medical Council Publications, London 2003 Tomorrow's Doctor.

[B14] Glaser BG, Strauss AL (1967). The Discovery of Grounded Theory: Strategies for Qualitative research.

[B15] McLachlan JC, McHarg J (2005). Ethical permission for the publication of routinely collected data. Medical Education.

[B16] Van Der Vleuten CPM (1996). The assessment of professional competence: Developments, Research and Practical Implications. Advances in Health Sciences Education.

